# Training-induced improvement in working memory tasks results from switching to efficient strategies

**DOI:** 10.3758/s13423-020-01824-6

**Published:** 2020-10-15

**Authors:** Tamar Malinovitch, Hilla Jakoby, Merav Ahissar

**Affiliations:** 1 Hebrew University of Jerusalem, Jerusalem, Israel; 2 Hadassah Academic College, Jerusalem, Israel

**Keywords:** Working memory, Cognitive training, Human memory and learning, Visual working memory

## Abstract

It is debated whether training with a working memory (WM) task, particularly *n*-back, can improve general WM and reasoning skills. Most training studies found substantial improvement in the trained task, with little to no transfer to untrained tasks. We hypothesized that training does not increase WM capacity, but instead provides opportunities to develop an efficient task-specific strategy. We derived a strategy for the task that optimizes WM resources and taught it to participants. In two sessions, 14 participants who were taught this strategy performed as well as fourteen participants who trained for 40 sessions without strategy instructions. To understand the mechanisms underlying the no-instruction group’s improvement, participants answered questionnaires during their training period. Their replies indicate that successful learners discovered the same strategy and their improvement was associated with this discovery. We conclude that *n*-back training allows the discovery of strategies that enable better performance with the same WM resources.

Working memory (WM) is defined as the ability to simultaneously retain and manipulate information within short time periods (Baddeley, 1992, 2003). The number of items that can be explicitly accessed and manipulated (i.e., WM capacity) is extremely limited and poses a strict bottleneck to human cognition (Cowan, 2001). Indeed, WM capacity is strongly correlated with fluid intelligence (Engle, Laughlin, Tuholski, & Conway, 1999; Süß, Oberauer, Wittmann, Wilhelm, & Schulze, 2002) and with academic achievements (Baddeley, 1992; Bayliss, Jarrold, Baddeley, & Gunn, 2005; Hitch, Towse, & Hutton, 2001; Swanson, 2004). One of the most studied WM tasks is the *n*-back task (e.g., Jaeggi, Buschkuehl, Jonides, & Perrig, 2008), in which participants are presented with a sequence of serially presented stimuli and are asked to respond when a stimulus is repeated at an interval of exactly *n* stimuli. This task requires holding the last *n* items, plus the new item, in WM. When each stimulus is presented, participants must compare it to their predicted target stimulus (the item presented *n* intervals earlier), respond if there is a match (target), and then update their WM representation to form a prediction for the next target stimulus. Since performance in this task is highly correlated with general intelligence scores, even compared with other WM tasks (Jaeggi, Buschkuehl, Perrig, & Meier, 2010), it has become a common task for training aimed at generally enhancing WM and fluid intelligence (e.g., Au et al., 2015; Redick, 2019; Schwaighofer, Fischer, & Bühner, 2015).

Training WM unequivocally yields improvement in the trained task, but the generalization of this benefit has been heatedly debated (e.g., Redick, 2019). Some meta-analyses and systematic reviews supported the existence of significant transfer (Au, Gibson, Bunarjo, Buschkuehl, & Jaeggi, 2020; Karbach & Verhaeghen, 2014). Yet others found no transfer to untrained tasks, or, at best, minimal transfer to very similar tasks (Au et al., 2015; Melby-Lervåg & Hulme, 2013; Melby-Lervåg, Redick, & Hulme, 2016; Redick, 2019; Soveri, Antfolk, Karlsson, Salo, & Laine, 2017). Gathercole, Dunning, Holmes, and Norris (2019) concluded that reliable transfer of WM training occurs only when the new task is very similar in structure (“near”) to the trained task and requires similar cognitive routines.

Others (e.g., Jacoby & Ahissar, 2013, 2015; Melby-Lervåg et al., 2016; Redick, 2019; Sala & Gobet, 2017; Simons et al., 2016) noted that “far” transfer is more characteristic of studies without an active-control group. In these studies, a no-contact control group that did not practice any task was included, either as the only control (e.g., Jaeggi et al., 2008) or as an additional control group, whose inclusion is crucial for attaining a significant transfer effect (e.g., Anguera et al., 2013). The no-contact group is not given monetary (or equivalent) rewards or stimulating personal attention, both of which positively impact performance. Therefore, differences in transfer may stem from the mere existence of a training protocol rather than from the specific training protocol of the experimental group (Foroughi, Monfort, Paczynski, McKnight, & Greenwood, 2016; Melby-Lervåg & Hulme, 2013; Shipstead, Redick, & Engle, 2012). Studies that used active control groups (trained with a similarly demanding task and a similar reward protocol) typically found either small near-only transfer (Linares, Borella, Teresa, Id, & Carretti, 2019) or no transfer at all (Jakoby, Raviv, Jaffe-dax, Lieder, & Ahissar, 2019).

The magnitude of transfer, when such was reported, is usually small, and is difficult to dissociate from a null result, since it is not resilient to correction for multiple comparisons. Typically, several tasks are assessed before and after training, and performance in most untrained tested tasks does not improve following training (Barnett & Ceci, 2002; Shipstead et al., 2012). Given that testing several tasks increases the probability of false positive(s), the target significance criterion should be raised (reviewed in Jacoby & Ahissar, 2013, 2015). However, the effect size of transfer to untrained tasks, if any, is small: ~0.3 standard deviations in methodologically weaker studies, ~0.01 in methodologically sound studies (Melby-Lervåg et al., 2016; Redick, 2019). Since the typical size of trained groups is also small (~15 per group; Chooi & Thompson, 2012; De Simoni & von Bastian, 2018; Gibson et al., 2012; Redick et al., 2013; Thompson et al., 2013), raising the target level of significance would have rendered the reported transfer nonsignificant (e.g., Anguera et al., 2013).

The combination of the huge effort required to conduct intensive training studies and the small (if any) generalization to untrained conditions that are not very similar, highlights the importance of understanding the cognitive mechanisms underlying training-induced behavioral improvement. Remarkably, these mechanisms have hardly been addressed. Deciphering these processes was the aim of the current study, with a specific focus on *n*-back training because it is the most commonly trained task. We asked, what is it that participants learn which enables their substantial improvement in a challenging updating task, designed to require limited-capacity online manipulations? The few recent studies that addressed this question suggest that the use of a task-specific strategy may facilitate training-induced improvement (Fellman et al., 2020; Laine, Fellman, Waris, & Nyman, 2018; Linares et al., 2019; Redick et al., 2013). Indeed, the importance of a strategy that reduces WM requirements has been gradually acknowledged (Redick, 2019). But can the use of an efficient strategy explain the entire learning process?

We began this study with practicing the *n*-back task ourselves and discussing our accumulative introspection of what facilitated our improved performance. These discussions clarified to us the strategy that we each had independently discovered. We measured whether participants who were explicitly taught this strategy could quickly reach the level of performance attained by those who go through intensive training without strategy instructions. We then deciphered, based on the self-reports of participants who had trained massively with no instructions, whether their improvement was associated with the discovery of an efficient (perhaps the same) strategy.

## Method

### The naïve strategy of *n*-updates versus the efficient strategy of 1-update

**Fig. 1:**
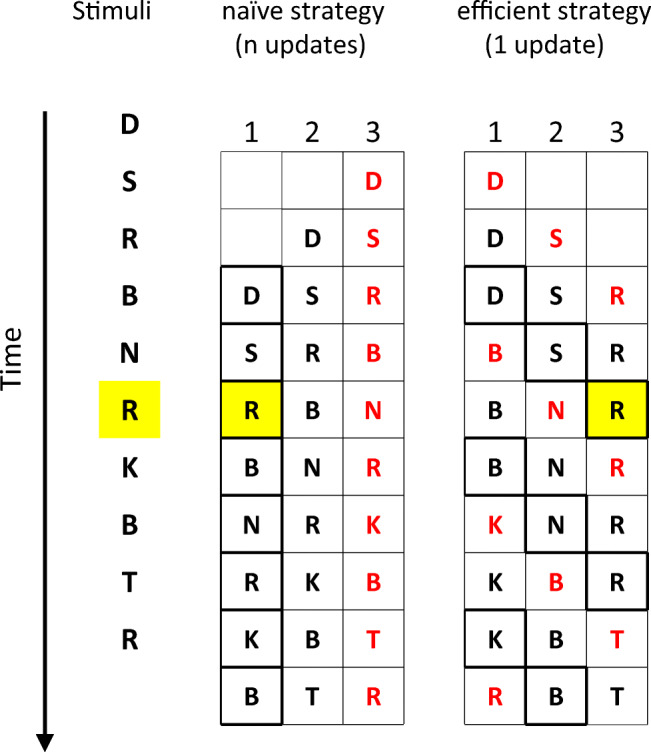
An illustration of the two strategies for *n*-back with letters: naïve *n*-updates (center) and an efficient 1-update (right), similar to that used by Laine et al. (2018), *n* = 3. The sequence of letters is shown in the left column. Each horizontal triplet of letters represents the information stored in WM during that trial before the letter on the left is presented (after the letter above was presented). The most recent letter in each trial is denoted in red. The slot storing the content that is being compared with the incoming letter is highlighted with a bold frame. Target stimuli repeated with an interval of 3 are highlighted in yellow. The naïve strategy stores the letters in WM in the order of their presentation, and each new letter is compared with the letter that is stored in the earliest memory slot. After each comparison, all three letters that are stored in WM are shifted one slot back (the earliest letter is discarded), and the new letter is inserted into the latest WM slot. Therefore, each step requires updating the content of three slots, as with a basic stack. By contrast, in the efficient 1-update strategy, only the attended slot is updated following each new stimulus, regardless of *n*. The attended position is shifted every step, but there is no change in the content of unattended positions. (Color figure online)

**Fig. 2:**
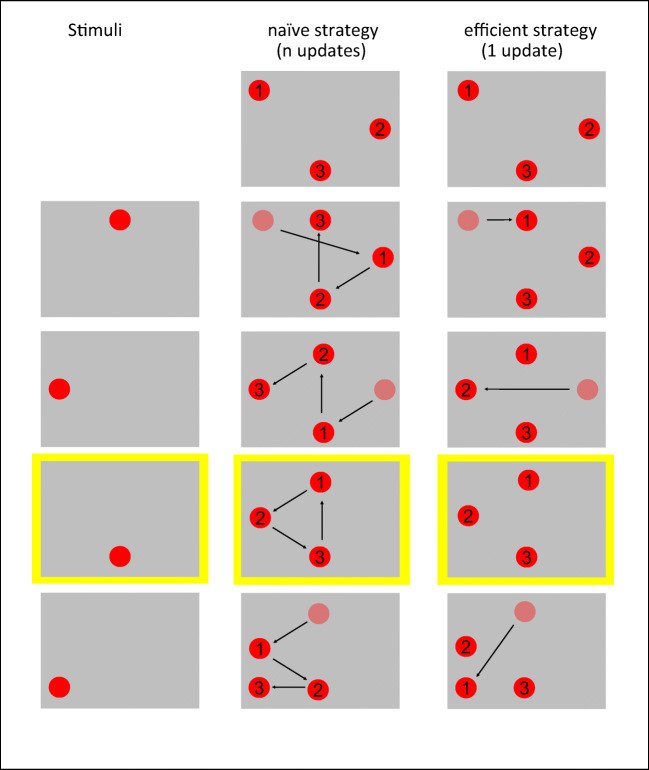
An illustration of two strategies for spatial *n*-back: naïve *n*-updates (center) and efficient 1-update (right), *n* = 3. The sequence of stimuli is presented in the left column. Each triplet of circles represents the information stored in WM during a trial, when a new circle is presented. Faded red circles represent the location of the oldest stimulus in WM, soon to be deleted. The arrows represent updates of locations in WM. Repetitions with *n* = 3 (targets) are highlighted in yellow. In the naïve strategy, locations are stored in WM in the order of their presentation. The “oldest” (presented *n* intervals earlier) item is compared with the newly presented item, and all three slots in WM are updated, each with the content of a more recent slot—as with a basic stack. In the efficient 1-update strategy, only one WM slot is compared and updated. What changes is the attended (and updated) slot in WM. Increasing *n* (see Fig. 5) increases the tracked loop with the number of retained positions, but not the number of updates per stimulus presentation. (Color figure online)

Naïve participants can typically perform well with *n* = 1 and *n* = 2, but find *n* ≥ 3 extremely challenging. The reason the task becomes difficult with *n* ≥ 3 is that participants need to update the content of *n* positions (slots) in WM following each item presentation. Figures 1 and 2 illustrate this naïve *n*-updates strategy (presented in the central column) for two types of *n*-back tasks—letters (Fig. 1) and spatial positions (Fig. 2). With this strategy, the last *n* items are always stored in WM in the order of their presentation. When a new stimulus is presented, it is compared with the oldest item (presented *n* stimuli earlier). Participants are asked to press a button if they recognize the match—stimulus repetition with an interval of *n* (denoted in yellow in Figs. 1 and 2). After each comparison, participants need to update all WM slots—all n(+1) items are “pushed” one position backward (left in Fig. 1), so that the most “recent” position holds the recently presented item and the “oldest” position holds the target of the next stimulus presentation. For example, when the items are letters, *n* = 3, the representation in WM is *D, S, R,* and the next letter is *B* (see Fig. 1, center, line 3)—this *B* will be compared with the item in the slot that holds the oldest item in WM—*D* (center, enclosed letter), and then added to WM at the most recent slot—following *R*. Then, the content of occupied slots in WM will be updated—shifted backwards, so that *D* will be dropped out, keeping the shifted three-letter representation—*S,R, B*. Thus, the naive strategy requires an update in the content of all WM slots—a shift in the slots of all *n* items in memory upon each stimulus presentation.

By contrast, the strategy described by Laine et al. (2018) for letters (see Fig. 1, right column) and its parallel for the spatial task that was derived by us (see Fig. 2, right column) includes no shifts in WM representation. Rather than shifting the items in WM slots, it shifts the slot to which attention is allocated in the given WM representation. The shift in the attended slot in WM does not put load on WM (Myers, Chekroud, Stokes, & Nobre, 2018). Crucially, in each trial only the item in the attended slot is updated (if it differs than the expected target). Thus, the strategy requires, at most, updating the content of one WM slot (compared with *n* slots in the naïve strategy), keeping track of which position is now relevant, and an attention shift, which does not occupy additional WM resources (Myers et al., 2018). For example, for letters and *n* = 3 (see Fig. 1, right), when the representation in WM is *D, S, R*, then *B* is the new letter, and the attended position is the first (line 3), only this position is updated so that the new representation in WM will be *B, S ,R*. When the next stimulus is presented (line 4), attention is shifted to the second position, and the new stimulus is compared with *S*, which will be updated if there is no match. Thus, if the new item is *N*, the updated WM sequence will be *B, N, R.* Next, the third position will be attended, and then back to the first (when *n* = 4, this loop has four positions, as illustrated in Fig. 5).

The difference between the amount of update required by each strategy can easily be seen when examining the similarity between consecutive WM representations, shown in consecutive lines in Fig. 1. One can also perceive the similarity in sound by sounding the preupdating and postupdating sequences (content of consecutive steps): *D, S, R* is much more similar to *B, S, R* (efficient 1-update strategy) than to *S, R, B* (naïve strategy), since the content of only one slot is modified in the former, as opposed to three slots in the latter.

The above description focuses on letters. We now extended the same conceptual strategy to other stimuli (though the analogy may not be transparent to participants). When the task is spatial (see Fig. 2), spatial locations of stimuli need to be retained in WM. Thus, the same *n*-updates versus 1-update strategy applies to the spatial task. In the naïve strategy, participants consistently compare the item in the first (oldest) slot, and then update the entire set of slots by pushing them backwards and removing the “oldest” slot from memory (as illustrated in Fig. 2, left). In the efficient strategy, only one slot is updated. This slot—the attended and updated one—changes with the presentation of each stimulus, in a loop with a length of *n* (for *n* items). Here too, efficiency results from solving the task using the 1-update strategy (only one WM slot is updated in each step of Fig. 2, right) and keeping track of which item needs to be attended next. As with letters, switching the strategy from updating all slots to updating only the attended slot, with the index looping over the number of items—first-second-third-first—reduces the WM resources required for attaining the same level of success.

In this study, we chose to use a spatial *n*-back task; in the past, we had trained a group of participants with this task, with no explicit strategy instructions, for 40 sessions (Jakoby et al., 2019). Most of those participants improved significantly in this task, but showed no transfer to other WM tasks. We now asked what these participants had actually learned during this training, and whether a similar degree of improvement could be gained in less time if participants were explicitly taught the efficient 1-update strategy.

### Experimental design and participants

In this paper, we compared the data of the two following groups:

1. The strategy-instruction group (*N* = 14), who received three training sessions—a naïve session with no strategy instructions, and two subsequent sessions. At the beginning of each of these two sessions, they watched a detailed 8-minute video clip with strategy instructions in Hebrew (the English version of this video clip can be found here [https://youtu.be/-21tuZQNMMQ]). Then an experimenter showed the participant a sequence of six stimuli and asked them to describe the representation in memory on Steps 4–6 when *n* = 3. The criterion for understanding was strategy-correct answers for all three steps, and it was met by all the participants. Participants were then asked to perform the task according to the strategy presented in the video clip. The interval between consecutive sessions was 1–8 days. Participants were told that the aim of the study is to assess how using this specific strategy affects their performance of the task. Data for this group were collected specifically for this study.
2. The no-instruction group, who trained for 40 sessions with no explicit strategy instructions (five times a week for 2 months). The data of this group have been previously published, in a study aimed at assessing transfer to other WM tasks, which found no transfer (Jakoby et al., 2019). Participants were told that the aim of the study is to assess how training for a task improves their performance in the trained task and in other memory-challenging tasks. Both groups answered the questionnaires detailed below, in which they expounded on the strategy they had used to perform the task.

The choice of 14 participants in the strategy-instruction group was aimed to match the number of participants who had previously formed the no-instruction group. In the context of this study, the relevant effect size is the magnitude of improvement in the trained task. Since improvement was larger than three standard deviations (Jakoby et al., 2019), 14 participants per group were sufficient in both the previously studied group (no-instruction) and the newly added one (strategy-instruction group). Statistical power analysis shows that to get the power of 0.9 with α = 0.01, based on the results of the first and the last session of the no-instruction group (Jakoby et al., 2019), at least 10 participants are needed per group (Table 1).

**Table 1:** Demographics of both groups, mean ± *SD*; the data of the no-instruction group have been published previously (Jakoby et al., 2019)

Group	Number (women)	Age (in years)	Years of education
Strategy-instruction	14 (9)	24.6 ± 2.1	14 ± 0.4
No-instruction	14 (11)	23.9 ± 3.8	15.7 ± 2.1

All participants received monetary compensation or course credit for their participation (for a detailed description of the monetary compensation of the no-instruction group, see Jakoby et al., 2019). The data of one participant from the no-instruction group were excluded from the analysis (data of 14 participants are reported) because her performance on the task before training was an extreme outlier (*z* score of over 2.5 in each session). Her first self-report indicates the discovery of the efficient strategy. We believe that she had discovered the strategy early in the first session. Importantly, all the reported results remain statistically significant when including this participant.

#### Spatial *n*-back task

**Fig. 3:**
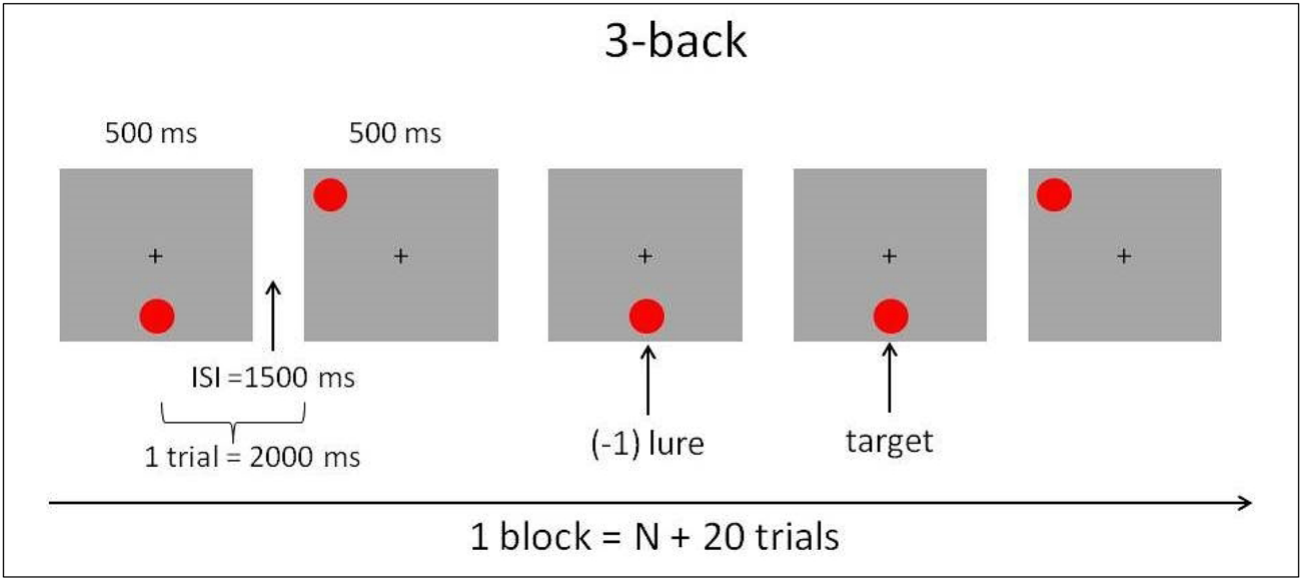
An illustration of five consecutive steps in a block of the spatial *n*-back task, *n* = 3

Both groups were administered the same spatial *n*-back protocol (Jakoby et al., 2019). In this protocol, red circles are presented sequentially, one circle every 2 seconds (stimulus duration 500 ms; interstimulus interval 1,500 ms), in one of eight positions on a virtual rectangle on a computer screen. Participants respond by pressing a space bar with their index finger whenever the location of a newly presented circle matches the location of the circle presented *n* steps back (target). No response is required for nontargets. Participants are notified about the relevant *n* at the beginning of each block. Each block comprises n+20 steps (stimuli) and includes six targets. Particularly confusing stimuli are lures: repetitions with an interval slightly different than *n*—a circle appears at a previous position (repetition) but with an interval of (*n* − 1) or (*n* + 1), as illustrated in Fig. 3. Differentiating lures from targets is difficult—participants tend to press the button upon detecting a repetition, even with different intervals (Duncan, 2003). In our experiment, we included three possible levels of lure difficulty: easiest—no lures, intermediate—four lures per block (two of each type), and most difficult—eight lures per block (four of each type). We included lures because it has been previously shown that lures increase WM load and the requirements of cognitive control (e.g., Redick & Lindsey, 2013; Szmalec, Verbruggen, & Kemps, 2011). Each block’s level of difficulty was determined as follows: If the participant’s performance was 85% correct or above (calculated as hit rate minus false alarm), the difficulty level for the following block was increased by adding four more lures. After reaching a level of eight lures in a block, reaching the 85% accuracy criterion increased *n* by one. When performance was 65% correct or below, the number of lures was decreased from eight to four to zero, and eventually *n* was decreased by one (and the next block, with the smaller *n*, would include eight lures). Difficulty level was not modified otherwise. Each session lasted ~30 min and consisted of 25 blocks with short breaks between them. The first two sessions began with *n* = 2 and four lures per block for all participants. Subsequent sessions began for each participant at the difficulty level they had reached during the last block of the previous session. The same protocol was administered to both groups.

##### Questionnaires

Both groups answered questionnaires regarding the strategies they had used to perform the task. In the strategy-instruction group, participants filled out questionnaires only at the end of the third session. First, they were asked to describe their strategy in their own words (i.e., explain what they had done and evaluate the efficiency of their strategy). Then, they were presented with illustrations of two strategies—the naïve *n*-updates strategy and the efficient 1-update strategy—and were asked to state which was closer to their own strategy (if any). This questionnaire had two goals: (1) to make sure that the participants in the strategy-instruction group had indeed used the explicitly taught strategy; (2) to see whether other methods were developed and used by the participants.

In the no-instruction group, each participant answered a questionnaire at the end of each week of training (five training sessions). The same questionnaire was administered every week. The questionnaire included two open question regarding strategy use (“In general, could you describe your strategy for performing the training task?” and “Is it a different strategy from the one that you used in last week’s training?”). The questions were open-ended and nonspecific so that no particular strategy would be implied, and no guidance would be inadvertently provided. The answers to all questionnaires were read and analyzed only after the experiment had ended, so that participants would not be affected by the experimenters’ expectations. To decide which strategy had been used and whether it was modified with training, we asked four independent reviewers, who were familiar with the task yet blind to participants’ performance, to evaluate based on each week’s reply of each participant whether she or he had used the efficient 1-update strategy, and if so.

## Results

### Improvement was substantially faster in the strategy-instruction group

Initial performance without instructions (performance during the first session, measured by mean *n* per session) did not differ between groups (strategy-instruction group: mean *n* = 2.65, *SD* = .37, 95% confidence interval (CI) [2.46, 2.84]; no-instruction group: mean *n* = 2.43, *SD* = .44, 95% CI [2.2, 2.66]; *p* = .38, in a two-tailed, two-sample unequal variance *t* test, Cohen’s *d* = .4). The second session began with an instructional video clip for the strategy-instruction group, and with no specific instructions in the no-instruction group. Afterwards, both groups performed the same task, with the same adaptive protocol (see Method section).

**Fig. 4:**
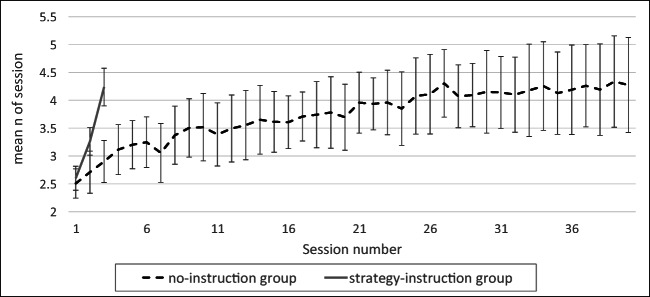
Performance as a function of session number in both groups. Mean *n* (~2.5) and error bars (CI 95%) were similar for both groups during the first session. Error bars of the two groups are similar in Session 1. The task is adaptive, which means that mean *n* increases as participants’ performance improves. Though both groups improved, the improvement rate was much faster in the strategy-instruction group. The mean *n* in the third session of the strategy-instruction group was similar to that attained by the no-instruction group after 25–40 sessions. The no-strategy instruction group showed greater cross-subject variability as training progressed, revealing increased variability in learning rate. A detailed analysis of individual learning variability is presented in Fig. 6

Mean performance in the second session significantly differed between the two groups, with the *n* of the strategy-instruction group (mean *n* = 3.25, *SD* = .43, 95% CI [3.02, 3.48]) being significantly higher than that of the no-instruction group (mean *n* = 2.56, *SD* = .58, 95% CI [2.36, 2.82]; *p* = .002, Cohen’s *d* = 1.29), as shown in Fig. 4.

In the third session, performance was very different between the groups (strategy-instruction: mean *n* = 4.3, *SD* = .66, 95% CI [3.95, 4.65]; no-instruction: mean *n* = 2.8, *SD* = .6, 95% CI [2.49. 3.11]; *p* < .00001, Cohen’s *d* = 2.36). In fact, within three sessions performance of the strategy-instruction group reached the level attained by the no-instruction group only after 25–40 sessions, and did not significantly differ from the no-instruction group’s final performance during the fortieth session (strategy-instruction group third session: mean *n* = 4.3, *SD* = .66, 95% CI [3.95, 4.65]; no-instruction group 40th session: mean *n* = 3.96, *SD* = 1.13, 95% CI [3.37, 4.55]; *p* = .46, Cohen’s *d* = .35).

A repeated-measures two-way analysis of variance (ANOVA) for the three first sessions (2 groups × 3 sessions( showed a significant main effect of session, *F*(2) = 62.99, *p* < .0001, η_p_^2^ = .83, indicating a general improvement as participants completed more sessions; a significant main effect of group, *F*(1) = 17.47, *p* < .0001, η_p_^2^ = .4, indicating different performance levels in the two groups, with the strategy-instruction group showing significantly better performance overall; and crucially—a significant interaction between session and group, *F*(2) = 26.7, *p* < .0001, η_p_^2^ = .68, indicating faster improvement in the strategy-instruction group.

**Fig. 5:**
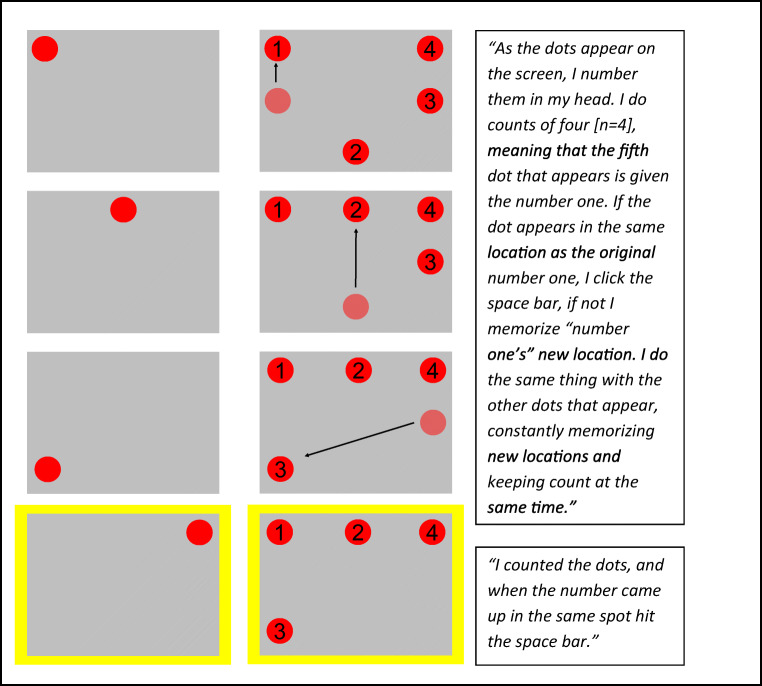
An illustration of the 1-update strategy for *n* = 4, based on participants’ reports. Arrows represent the updated location; faded red circles represent the attended (oldest) position in WM, soon to be forgotten; a trial with a match (target) is marked in yellow. (Color figure online)

Figure 4 plots mean performance (mean *n* per session) as a function of session number in the two groups. The initial and end points are similar, but the strategy-instruction group improved much faster. Cross-participant variability is similar for both groups in the first session (strategy-instruction *SD* = .37, no-instruction *SD* = 0.44 in the no-instruction group), and it increases for both groups during training, with a greater increase observed in the no-instruction group (strategy-instruction *SD* = .66, no-instruction *SD* = 1.13). This pattern results from the substantially different rates of improvement across participants, particularly when no explicit instructions are given. Large cross-participant variability was also observed in previous (no-instruction) training studies (e.g., Jaeggi, Buschkuehl, Jonides, & Shah, 2011). Previously, this variability was attributed to the extent to which general WM capacity increased. However, Figs. 4 and 5 show that this cross-participant variability results from different success rates in discovering the efficient task-specific 1-update strategy.

### Improvement in the no-instruction group is associated with the discovery of the efficient strategy

Participants in the strategy-instruction group indicated in the questionnaires that they all understood and used the instructed strategy during the two postinstruction sessions.

**Fig. 6:**
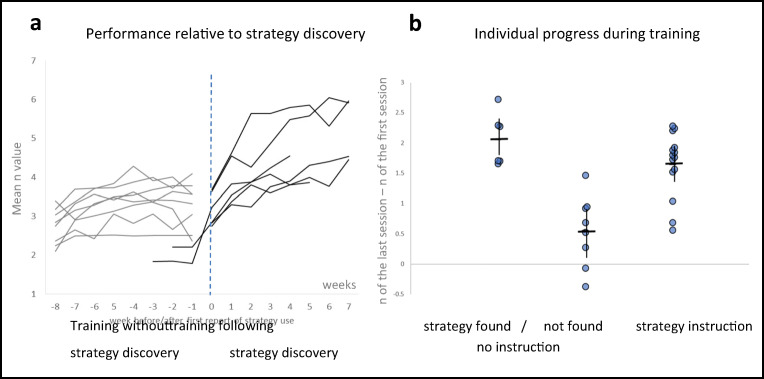
Individual learning trends, **a** Individual learning curves of training participants as a function of training weeks. The first point of each plot represents the number of weeks without (before any) use of the efficient strategy. Week 0 is the week at which the participant explicitly reported using the strategy for the first time. Individuals who begin at −8 are those who did not report the use of the efficient strategy throughout their 8 training weeks (indicated by gray lines). Black lines represent participants who unequivocally reported the use of the strategy at some point. Performance in each week is denoted by the mean level of *n* achieved by the participant in this week. Explicit reports of the strategy are associated with a sharp rise in the performance curve. **b** Individual gains in mean-session *n* during training (difference between the last and first sessions) for participants in both groups. Reference lines represent group means and CI of 95%. Participants in the no-instruction group are divided into two subgroups—those who unequivocally discovered and used the efficient strategy, and those who did not report the strategy. Changes in *n* of individuals in the no-instruction group range between −0.376 and 2.72, while in the strategy instruction group, all changes are positive. Importantly, within the no-instruction group all individuals whose reports indicate a discovery of the efficient strategy improved more than all individuals who did not report such a discovery

Analyzing the self-reports of participants in the no-instruction group was complex, because the verbal reports of eight (out of 14) participants were too vague to determine or rule out any specific strategy. However, six (out of 14) participants explicitly indicated that they used the efficient 1-update strategy starting from a particular training week (see Fig. 6). For example: “As the dots appear on the screen, I number them in my head. I do counts of four [*n* = 4], meaning that the fifth dot that appears is given the number one. If the dot appears in the same location as the original number one, I click the space bar, if not I memorize “number 1s” new location. I do the same thing with the other dots that appear, constantly memorizing new locations and keeping count at the same time.” Figure 5 illustrates how this account directly maps to the implementation of the efficient 1-update strategy with *n* = 4.

Figure 6a plots performance as a function of training week for each of the 14 participants of the no-instruction group. Performance is plotted with respect to “Week 0”—the week in which they discovered the efficient strategy, as evident from their self-report questionnaire (submitted at the end of every training week). As described above, the plots of six participants either started at or crossed Week 0. Four of them discovered this strategy within their first training week—that is, within their first five sessions (and hence their plots begin at “0”)—and the other two discovered it later, one in the third week and the other in the fourth week. Their slopes abruptly rise following this discovery. Plots of participants who did not report discovering a strategy during their 8 training weeks begin at “−8”, indicating that they trained 8 weeks without discovering the efficient strategy.

Figure 6b shows the individual gains in *n* between the first and last sessions in the strategy-instruction group and in two subgroups of the no-instruction group—the six participants who explicitly deciphered the efficient strategy, and the eight who did not. All participants improved in the strategy-instruction group, though not to the same extent. In the no-instruction group, improvement differed significantly between those who explicitly deciphered the efficient strategy and those who did not (*p* = .00067 in a Mann–Whitney *U* test), with no overlap between the degree of improvement of individuals in the two subgroups. Thus, the high cross-participant variability in the no-instruction group (see Fig. 4) is largely explained by the difference in improvement between those who discovered the efficient strategy, and substantially improve, and those who did not, whose improvement ranges between small to none at all.

## Discussion and conclusions

Our results indicate that training-induced improvement in the n-back task can be fully explained by the discovery of a task-specific strategy. Hence, improvement does not indicate a general enhancement of WM capacity. This finding explains previous findings of no transfer, or only very-near transfer to other types of the *n*-back task (e.g., Jakoby et al., 2019; Linares et al., 2019). Based on our results, we predict that performance in untrained tasks will improve only when the discovered efficient strategy applies, and its relevance is transparent to the participants. Thus, performance in tasks with the same structure may be improved (Fellman et al., 2020; Redick, 2019), as previously reported (Linares et al., 2019). However, performance in most other WM tasks, and even in *n*-back tasks for which the strategy is difficult to implement (Jakoby et al., 2019), is not expected to benefit from the discovery of this strategy. This account is in line with the proposal of Gathercole et al. (2019), who claim that training-induced transfer occurs only when participants have acquired a new complex cognitive skill during training, and when that skill can be applied to a novel task.

Though to the best of our understanding, the same efficient strategy was repeated across participants, we do not claim that this is the only possible strategy. Yet, theoretically, we expect all efficient strategies across WM tasks to have something in common. In fact, and as mentioned before, our strategy is the same or very similar to the strategy described previously for *n*-back with letters (Laine et al., 2018) and with digits (Fellman et al., 2020) in their studies characterizing the continuous effect of training with and without an explicit strategy. We assert that all efficient strategies reduce the number of manipulations in WM per trial, compared with the naïve strategy. Importantly, there is no need to reduce the total number of operations per trial—only the operations that put load on WM. For example, scanning through items without changing their slot in WM does not add to WM load (Myers et al., 2018).

This training study provides further support for the strategy mediation theory of WM improvement (Dunning & Holmes, 2014; Peng & Fuchs, 2017), compared with the capacity theory (Engle & Kane, 2003). The strategy mediation theory assumes that WM has a relatively fixed capacity, and therefore claims that WM performance is determined by the efficiency with which its capacity is used (Bailey, Dunlosky, & Kane, 2008; McNamara & Scott, 2001). The capacity theory assumes that capacity can increase with practice, and is often described using the muscle-like metaphor—efficient training strengthens WM by increasing capacity (described by Peng & Fuchs, 2017).

Yet most of the literature of strategy mediation theory does not specify the strategies that would be efficient for WM tasks. The description of rehearsal or chunking (McNamara & Scott, 2001; Peng & Fuchs, 2017; Turley-Ames & Whitfield, 2003) does not capture the unique structure of WM tasks, which are designed to require online manipulations that cannot be organized into fixed chunks, or at least not in any straightforward manner. Our study adds to recent studies (Fellman et al., 2020; Laine et al., 2018) in specifying an efficient strategy for the case of the spatial n-back task. Unlike *n*-back tasks that use items that can be easily named, like letters or digits, spatial tasks cannot benefit from subvocal rehearsal of the items (see Chooi & Logie, 2020). Still, subvocal verbalization can help memorizing which of the *n* items in WM should be updated in each step.

The positive impact of strategy instructions had been recently studied. For example, Fellman et al. (2020) taught participants a specific strategy for the *n*-back task with digits via the Internet, and found that using this strategy facilitated initial learning. However, the advantage of explicit instruction, observed during the first few sessions, was partial, as the strategy group continued to improve throughout the 12 training sessions, and performance of the tutored and nontutored groups was similar following the first few training sessions. Thus, instruction was beneficial, but its impact was smaller than in our case, perhaps due to the nature of Internet training, or due to the difference in task stimuli. Another potential contribution to the enhanced efficiency of instruction in our study, which yielded the equivalent of more than 25 uninstructed training sessions following only three sessions, was the effort we put on strategy clarity (presenting instructions with a video clip [https://youtu.be/-21tuZQNMMQ]), and verification of participants' understanding. Additionally, we showed that the large individual differences in training-induced improvement within a no-strategy group delineate the participants who discovered an efficient strategy spontaneously versus those who did not.

One of the most interesting questions that our results have raised is what differentiates people who develop an efficient strategy during training, even without explicit instructions, from those who do not. Studying this question systematically requires testing whether those who developed an efficient strategy for one task also tend to develop efficient strategies for other challenging tasks, which is beyond the scope of this study. There is some evidence that individuals with larger WM pretraining are the ones who benefit more from training (Foster et al., 2017; Redick, 2019; Wiemers, Redick, & Morrison, 2019). There may be a link between initial WM capacity and the ability to quickly and efficiently adapt a strategy for a task, or there may be another cognitive trait underlying both. We do not find evidence for that in our group of participants; performance during the first session is not a good predictor of learning rate, though perhaps mean performance during the first session already includes some learning. Another suggestion in the literature is that action video game players are more likely to find efficient strategies, as their ability to learn is enhanced (Bejjanki et al., 2014, Green & Bavelier, 2014), perhaps due to enhanced attention and spatial cognition (Bediou et al., 2018). This claim had been challenged, and the findings regarding the advantages of strategic video games have been questioned (e.g., Roque & Boot, 2018; Sala, Tatlidil, & Gobet, 2018). We should note that even if action video game players demonstrate better strategies, it is still not clear whether playing action video games is the reason or the result of this enhanced strategic ability (or both).

Finally, perhaps the most important conclusion of this study is its contribution to the accumulative recent body of research, which support the strategy account of WM training, and indicate that time has come to change the metaphors we use to describe WM training studies. Training WM does not open a common bottleneck. Successfully trained individuals do not perform the same operations faster or better. They change the set of operations used to solve the task. This type of change is likely to underlie the acquisition of all expertise. When the same operations are administered to the same sequences of stimuli repeatedly, as in word reading, we replace the WM operations with chunking and schemas. But when the crux of the task is using the operations on untrained stimuli sequences (as in reading nonwords), chunking cannot replace online computations. Hence, training-based improvement probably results from using a set of more efficient task-specific operations. Better understanding of these task-specific strategies may both teach us about the structure of WM and facilitate performance in tasks that heavily load on our limited WM resources.
